# Matured hop extract reduces body fat in healthy overweight humans: a randomized, double-blind, placebo-controlled parallel group study

**DOI:** 10.1186/s12937-016-0144-2

**Published:** 2016-03-09

**Authors:** Yumie Morimoto-Kobayashi, Kazuaki Ohara, Hiroshi Ashigai, Tomoka Kanaya, Kumiko Koizumi, Fumitoshi Manabe, Yuji Kaneko, Yoshimasa Taniguchi, Mikio Katayama, Yasuyuki Kowatari, Sumio Kondo

**Affiliations:** 1 Research Laboratories for Health Science and Food Technologies, Kirin Company, Ltd, 1-13-5, Fukuura, Kanazawa-ku, Yokohama, 236-0004 Japan; 2 Central Laboratories for Key Technologies, Kirin Company, Ltd, 1-13-5, Fukuura, Kanazawa-ku, Yokohama, 236-0004 Japan; 3 Ueno Clinic, Aiseikai Public Interest Incorporated Foundation, 2-18-6, Higashiueno, Taito-ku, Tokyo, 110-0015 Japan; 4 Fukushima Healthcare Center, Kensyokai Medical Corporation, 2-12-16, Tamakawa, Fukushima-ku, Osaka 553-0004 Japan

**Keywords:** Matured hop extract, Matured hop bitter acids, Obesity, Body fat

## Abstract

**Background:**

Hops are the main components of beer that provide flavor and bitterness. Iso-α-acids, the bitter components of beer, have been reported to reduce body fat in humans, but the bitterness induced by effective doses of iso-α-acids precludes their acceptance as a nutrient. The matured hop bitter acids (MHBA) of oxidized hops appear to have a more pleasant bitterness compared to the sharper bitterness of iso-α-acids. While there has been little information concerning the identity of the MHBA compounds and their physiological effects, MHBA was recently found to be primarily composed of oxides derived from α-acids, and structurally similar to iso-α-acids. Here, we investigated the effects of matured hop extract (MHE) containing MHBA on reducing abdominal body fat in healthy subjects with a body mass index (BMI) of 25 to below 30 kg/m^2^, classified as “obese level 1” in Japan or as “overweight” by the WHO.

**Trial design:**

A randomized, double-blind, placebo-controlled parallel group study.

**Methods:**

Two hundred subjects (male and female aged 20 to below 65 years with a BMI of 25 or more and less than 30 kg/m^2^) were randomly assigned to two groups. During a 12-week ingestion period, the subjects in each group ingested daily 350 mL of test-beverage, either containing MHE (with 35 mg MHBA), i.e. the namely active beverage, or a placebo beverage without MHE. The primary endpoint was reduction of the abdominal fat area as determined by CT scanning after continual ingestion of MHE for 12 weeks.

**Results:**

Compared to the placebo group, a significant reduction was observed in the visceral fat area after 8 and 12 w, and in the total fat area after 12 w in the active group. There was also a concomitant decrease in body fat ratio in the active group compared to the placebo group. No adverse events related to the test beverages or clinically relevant abnormal changes in the circulatory, blood and urine parameters were observed in either group.

**Conclusions:**

The present study suggests that continual ingestion of MHE safely reduces body fat, particularly the abdominal visceral fat of healthy overweight subjects.

**Trial registration:**

UMIN-CTR UMIN000014185

**Electronic supplementary material:**

The online version of this article (doi:10.1186/s12937-016-0144-2) contains supplementary material, which is available to authorized users.

## Introduction

Obesity increases the risk of metabolic disorders, such as insulin resistance, hyperlipidemia and hypertension, which are risk factors of cardiovascular disease [1–3]. Because excess energy intake is a key cause of obesity, suppression of energy intake and increase of energy expenditure are obvious therapeutic approaches. Since the prevalence of obesity is rapidly increasing around the world, intensive research has been conducted into the development of drugs and functional foods which can prevent obesity. Indeed, some natural products have been reported to decrease body fat in humans, while for others there is little clinical evidence to demonstrate their anti-obesity effect [4, 5].

Hops, the immature inflorescences of the female hop plant (*Humulus lupulus* L.), have been widely used to add flavor and bitterness to beer. Iso-α-acids, major bitter components in beer, are converted from α-acids in hops by isomerization during the brewing process [6], and have been reported to have many health benefits [7–9]. Among these, iso-α-acids were shown to prevent obesity in mice and humans [10, 11]. However, it is difficult to add an effective dose of iso-α-acids to foods because of their bitterness. Interestingly, during storage the α- and β-acid content in hops decreases, and other bittering components accumulate [12]. There is little information concerning the identity of these compounds mainly because of their structural complexity and difficulty in handling. Recently, it has been found that these components, called matured hop bitter acids (MHBA), primarily consist of α-acid oxides, which possess a common β-tricarbonyl moiety in their structures similar to α-, β- and iso-α-acids [13–16]. In addition, a recent study suggested that the oxidation products of α-acids may result in a more pleasant tasting bitterness compared to the sharper bitterness of iso-α-acids [17]. On the basis of these findings, MHBA may be more useful for food applications compared to iso-α-acids.

It has also been reported that MHBA ameliorates diet-induced body fat accumulation in rodents by enhancing thermogenesis in brown adipose tissue (BAT) *via* sympathetic nerve activity innervating BAT [18]. BAT is a major organ for cold- and diet-induced adaptive thermogenesis [19]. It has long been thought that BAT is abundant in rodents, but absent or negligible in adult humans. However, recent studies using fluorodeoxyglucose positron emission tomography (FDG-PET) in combination with computed tomography (CT) revealed that adult humans have metabolically active BAT [20]. The prevalence and activity of BAT in the human body are inversely related to body fat content [21]. These studies suggest that regulation of BAT activity is a possible therapeutic target for obesity in humans. Thus, it might be expected that MHBA reduces body fat in humans, however, there is no clinical evidence to support these effects of MHBA in humans. To examine the effects of MHBA on reducing body fat in humans, we prepared matured hop extract (MHE), a hot water extract from oxidized hops, which includes 18.3% of MHBA and undetectable amounts of α-, β- and iso-α-acids, in the solid content.

In this study, we investigated the effects of 12-week ingestion of MHE on body fat reduction in healthy subjects with a BMI of 25 or more and less than 30 kg/m^2^ by a randomized, double-blind, placebo-controlled parallel group study. In addition, we evaluated the safety of MHE ingestion.

## Materials and methods

### Study procedures

The protocol (Protocol No. 26920) was approved by the Institutional Review Boards of Ueno Clinic, Aiseikai Public Interest Incorporated Foundation (Tokyo, Japan) and Kensyokai Medical Corporation (Osaka, Japan) in accordance with the ethical standards established in the Helsinki Declaration and the ethical guidelines for epidemiological research of the Ministry of Education, Culture, Sports, Science and Technology, and the Ministry of Health, Labor and Welfare of Japan. This study was registered with the UMIN Clinical Trials Registry as UMIN000014185 (Title; Study of effects of food containing hop extract on reducing body fat), and was conducted in compliance with the protocol. Written informed consent was obtained from all subjects. This study was performed by a contract research organization, TTC Co., Ltd. (Tokyo, Japan) from May 2014 to December 2014 at the following three facilities: Ueno Clinic, Aiseikai Public Interest Incorporated Foundation (Tokyo, Japan), Shinjyuku Oiwake Clinic, Seikokai Medical Corporation (Tokyo, Japan), and Fukushima Healthcare Center, Kensyokai Medical Corporation (Osaka, Japan).

### Subjects

The subjects were male and female aged 20 to below 65 years with a BMI of 25 or more and less than 30 kg/m^2^. A BMI of 25 to below 30 kg/m^2^ is defined as “obese (level 1)” by the Japan Society for the Study of Obesity and as “overweight” by the WHO. The exclusion criteria were as follows: on a diet; use of oral medication affecting body fat or lipid metabolism; constant use of dietary supplements or functional foods affecting body fat or lipid metabolism; constant ingestion of foods enriched with hop constituents; excessive alcohol-drinking behavior; under treatment; onset of possible allergy symptoms; with a history of serious disease (e.g., diabetes, liver disease, kidney disease or heart disease), thyroid gland disease, adrenal gland disease, or other metabolic disorder; with severe anemia; with a history of digestive disease affecting digestion or absorption; under treatment or with a history of drug addiction or alcoholism; possible pregnancy, pregnancy or lactating; unfavorable results of the given lifestyle questionnaire or blood test; participation in a clinical study within the last month prior to this study or possible participation in another clinical study; donation of over 200 mL of blood or blood component within the last month prior to this study or over 400 mL of blood or blood component within the last three months prior to this study; any other reason for ineligibility as judged by the site investigators.

### Target sample size

Based on a preliminary human study, MHE (with 35 mg MHBA) was expected to reduce body fat by 5.8 cm^2^ with a standard deviation of 12.1 cm^2^ compared to the placebo group. Setting the significance level at 5% by an unpaired Student's *t*-test and the power at 0.85, the number of subjects required per group was estimated to be 80. To account for an expected dropout rate of 20%, the number of subjects required per group was estimated to be 100.

### Preparation of MHE

Hop pellets were purchased from HopSteiner (Mainburg, Germany). Tricyclooxyisohumulones A was prepared as described [14]. Hop pellets (300 g) were stored at 60°C for 120 h to oxidize α- and β-acids and then extracted with pre-warmed H_2_O (50°C, 3 L) for 1 h. During extraction, the temperature of the extract was maintained at 50°C. The extract was filtered to remove the debris and then treated with activated charcoal and PVPP for 2 h at room temperature. After treatment, the mixture was filtered to separate the extract from activated charcoal and PVPP. After concentration to 11-12° Brix, the filtrate was heated to 90°C for 4 h and then cooled to room temperature to yield a pale brown liquid (450 g) referred to as MHE. MHE contains 12% solid content, which consists for 18.3% of MHBA according to HPLC analysis using a previously reported method, while the amounts of α-, β- and iso-α-acids were undetectable [16].

### Test beverages

We prepared 350 mL of test beverages, either containing MHE (with 35 mg MHBA) in the case of the active beverage, or without MHE for use as the placebo. The nutritional composition of the test beverages is shown in Table 1. The controller confirmed that there were no discernible differences between the appearance and taste of the two test beverages, and a record was kept before allocation and at the blind broken.

**Table 1:** Nutritional compositions of test beverages (per 100 mL)

	Active beverage	Placebo beverage
Energy (kcal)	4.6	4.2
Protein (g)	ND	ND
Lipid (g)	ND	ND
Available carbohydrate (g)	0.4	0.3
Dietary fiber (g) (resistant maltodextrin)	1.5	1.5
MHE (mg, as the amount of MHBA)	10	0

ND, not detectable

### Study design

A randomized, double-blind, placebo-controlled parallel group study was conducted over 18 weeks, consisting of a 2-week pre-ingestion period (−2 to 0 w), a 12-week test beverage ingestion period (0 to 12 w), and a 4-week follow-up period without test beverage ingestion (12 to 16 w). Subjects were screened for eligibility over the 2 weeks preceding the ingestion period, and visited at 0, 4, 8, 12 and 16 w for the test. Interview, measurement of anthropometric and circulatory parameters, blood sampling, and urine sampling were conducted at each test. CT scanning was performed at 0, 8, 12 and 16 w. Only at the screening test, a lifestyle questionnaire was answered by all subjects, and a pregnancy test was taken by the female subjects.

The controller randomly assigned the subjects in a 1: 1 ratio to two groups with random numbers, and stored an assignment list in a sealed container until database lock. Throughout the study, the subjects, all investigators and study personnel except for the controller remained blinded. During the ingestion period each subject took a test beverage 1 time per day. The time of test beverage ingestion was not limited except on test days, when subjects ingested the test beverage after the test was completed. Subjects were instructed to continue their usual eating, exercise, sleeping, smoking and drinking habits, and to avoid overdrinking throughout the study. Use of oral medication, dietary supplements and functional foods affecting body fat or lipid metabolism, and foods enriched with hop constituents were prohibited. On the day before a test, subjects were prohibited drinking alcohol and had to finish their evening meal by 22:00. Since then, eating and drinking (except for water) were prohibited until the test on the following day was completed. On the test day itself, smoking was prohibited until the test was completed.

Discontinuance criteria of the study for the subjects were as follows: risk of the subject′s safety; difficulty of continuation of the study due to a serious adverse event or accident; continuous or serious non-compliance with the protocol by the subject; pregnancy; any other reason for discontinuation as judged by the site investigators.

### Measurements of anthropometric and circulatory parameters

Height was measured only at the screening test. Body weight, body fat ratio, waist circumference, hip circumference, systolic blood pressure (SBP), diastolic blood pressure (DBP) and pulse rate were measured at each test. The BMI was calculated from height and body weight, and the ratio of waist to hip circumferences was calculated. Blood pressure was measured once after a 5-min rest with the subject in the sitting position. The body fat ratio was measured by bioelectrical impedance analysis (BIA) using an Inner Scan 50V BC-621 (Tanita, Tokyo, Japan). Inner Scan 50V BC-621 is a single-frequency BIA device that uses eight polar electrodes and is based on reactance technology, a method that measures the body fat ratio more accurately than the conventional BIA method by adopting reactance values in addition to impedance values. An algorithm incorporating impedance, age and height is used to estimate the body fat ratio. The body fat ratio measured by the BIA system with eight polar electrodes has been reported to correlate with that measured by the dual energy X-ray absorptiometry (DXA) method [22].

### Measurement of abdominal fat areas

The subjects underwent CT scanning of the abdominal transverse section at the umbilical position using a Robusto-Ei (Hitachi Medico, Tokyo, Japan) in Shinjyuku Oiwake Clinic and an Asteion Super4 Edition TSX-021B (Toshiba Medical Systems, Tochigi, Japan) in the Fukushima Healthcare Center. The visceral fat area (VFA), subcutaneous fat area (SFA) and total fat area (TFA) were calculated from CT image using fatPointer software (Hitachi Medico) in the Shinjyuku Oiwake Clinic and SlimVision5 software (Cybernet Systems, Tokyo, Japan) in the Fukushima Healthcare Center.

### Blood chemistry and hematological examinations

The concentrations of the following parameters were measured in fasting blood samples: total protein (TP), albumin (Alb), total bilirubin (T-Bil), aspartate aminotransferase (AST), alanine aminotransferase (ALT), lactate dehydrogenase (LDH), alkaline phosphatase (ALP), γ-glutamyl transpeptidase (γ-GTP), total cholesterol (TC), low-density lipoprotein cholesterol (LDL-C), high-density lipoprotein cholesterol (HDL-C), triglyceride (TG), phospholipid (PL), free fatty acid (FFA), glucose, uric acid (UA), blood urea nitrogen (BUN), creatinine (Cre), sodium (Na), chloride (Cl), potassium (K), calcium (Ca), magnesium (Mg), iron (Fe), total iron-binding capacity (TIBC), unsaturated iron-binding capacity (UIBC) and ferritin for blood chemistry; and white blood cell count (WBC), red blood cell count (RBC), hemoglobin (Hb), hematocrit (Ht) and platelet count (Plt) for hematology. All blood analyses were performed by a commercial clinical laboratory (BML, Inc., Tokyo, Japan) using the automatic analyzers LABOSPECT008K (Hitachi High-Technologies, Tokyo, Japan) for blood chemistry and XE-5000 (Sysmex, Hyogo, Japan) for hematology.

### Urinalysis

Qualitative urinalyses of protein, glucose, occult blood, urobilinogen and keton bodies were performed using fasting urine samples. Urine pH was also measured. All urinalyses were performed by BML, Inc. using the automatic analyzer AX-4280 (Arkray, Kyoto, Japan).

### Record of diet and physical activity, and daily life diary

Subjects recorded the contents of their daily meals, snacks and beverages except for water, as well as the number of steps taken by pedometer, on a paper record of dietary and physical activity for three days before each test at 0, 4, 8, 12 and 16 w. Daily average values for dietary composition (energy, protein, fat, carbohydrate and dietary fiber) were determined from the dietary record using Calorie Checker version 5 (Healthcare Total Solutions, Tokyo, Japan) by a nutritionist, and daily average number of steps taken was calculated as physical activity. The subjects also recorded the test beverage ingestion, subjective symptoms, and daily activities, including eating habits, exercise habits, alcohol intake and drug intake in the diary every day.

### Interview

A physician interviewed the subjects about their physical condition and subjective symptoms.

### Endpoints

As determined before the beginning of the study, the primary endpoint was reduction of the abdominal fat area after continual ingestion of the active beverage for 12 weeks, and secondary endpoints were a decrease of body weight, BMI, body fat ratio, waist circumference and hip circumference. Safety endpoints were occurrence of adverse events and clinical laboratory parameters (body weight, circulatory parameters, blood chemistry parameters, hematological parameters, and urinalysis parameters). When an adverse event occurred, the site investigators conducted a follow-up survey until disappearance of the symptoms or a trend of recovery from the event had set in (Last date of the follow-up survey: December 8, 2014).

### Statistical analysis

Data were expressed as the means (SEM). A paired Student′s *t*-test was used to evaluate for each group the change in values from when test-beverage ingestion began (0 w), and an unpaired Student′s *t*-test was used to compare differences between the two groups at each test point. In addition, for primary and secondary endpoints, the effects of two factors (time and group) and a time-by-group interaction were analyzed by two-way analysis of variance (ANOVA) using values obtained during the test-beverage ingestion period. Differences were considered significant at *P* < 0.05. All statistical analyses were performed using PASW statics 18 (IBM, Armonk, NY) or Microsoft Excel 2010 (Microsoft, Redmond, WA).

## Results

### Background of the subjects

**Fig. 1:**
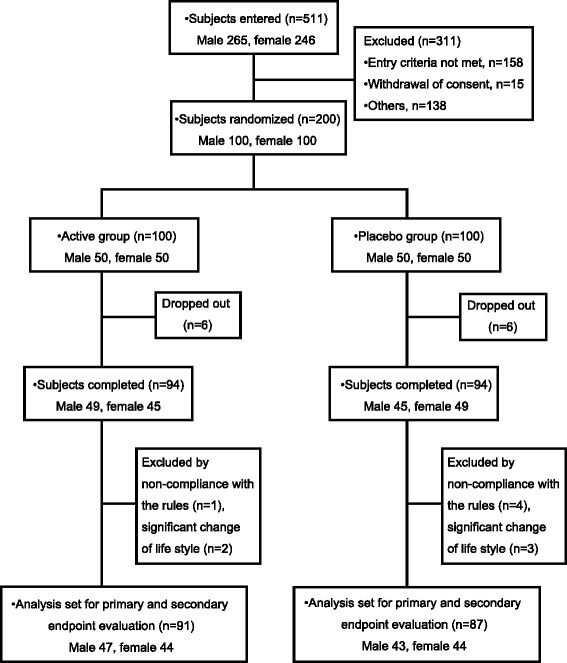
Flow diagram of the progress stages of this study

The subjects were recruited from April 28, 2014 to June 8, 2014. The disposition of the subjects enrolled in this study is shown in Fig. 1. Excluding 311 of the 511 participants in the screening test in accordance with the inclusion and exclusion criteria, 200 subjects (male = 100, female = 100) were judged eligible by the site investigators, and were randomized into the active and the placebo group (100 subjects per group). Twelve subjects dropped out of the study because of personal problems not related to the study. In accordance with the exclusion criteria, among the 188 remaining subjects who finished the study (94 subjects in each group), 10 subjects were excluded from primary and secondary endpoint evaluation by the site investigators before release of the double-blind due to their failure to obey compliance rules (five subjects) or because of a significant change of life style that affected the study (five subjects). Consequently, the statistical analyses for primary and secondary endpoint evaluation were conducted on 178 subjects (91 subjects in the active group and 87 subjects in the placebo group). The baseline characteristics of the subjects measured at the screening test are shown in Table 2. No significant differences were found for any parameter before the two groups were given the test beverages. Average intake rates of test beverages during the ingestion period in the active group and the placebo group were 99.0 ± 2.2% and 99.0 ± 2.1%, respectively. Table 3 shows daily average values for parameters linked to dietary composition and physical activity. There were no significant differences between the two groups in any of these parameters throughout the study, although the daily average energy intake and dietary fiber in the active group appeared to be significantly decreased after, respectively, 16 w and 4 w of test-beverage ingestion.

**Table 2:** Baseline characteristics of the subjects

Parameter	Active group	Placebo group
n	91	87
Age (years)	44.8 (1.2)	44.1 (1.2)
Height (cm)	164.36 (0.93)	164.18 (0.90)
Body weight (kg)	74.65 (0.88)	74.42 (0.87)
BMI (kg/m^2^)	27.57 (0.13)	27.54 (0.12)
Body fat ratio (%)	32.12 (0.79)	32.15 (0.78)
Waist circumference (cm)	94.15 (0.55)	93.19 (0.52)
Hip circumference (cm)	101.09 (0.45)	101.08 (0.40)
Waist/Hip	0.932 (0.005)	0.922 (0.004)

Data are expressed as means (SEM). No significant differences were observed in any parameters between the two groups

**Table 3:** Dietary composition and physical activity

Parameter	Group	Ingestion period				Follow-up period
		0 w	4 w	8 w	12 w	16 w
Energy (kcal/day)	A	1810.1 (50.9)	1821.2 (48.9)	1756.8 (44.3)	1774.2 (42.8)	1728.5 (46.3)^*^
	P	1795.2 (38.3)	1781.6 (48.9)	1782.0 (43.9)	1803.5 (42.8)	1769.7 (45.7)
Protein (g/day)	A	64.07 (1.98)	65.28 (2.06)	63.26 (1.71)	65.25 (1.70)	62.30 (1.99)
	P	63.63 (1.67)	63.51 (2.10)	64.71 (1.85)	64.09 (1.66)	65.13 (2.07)
Fat (g/day)	A	62.03 (2.12)	65.66 (2.19)	62.45 (2.36)	63.36 (1.94)	59.14 (2.30)
	P	65.00 (2.06)	62.53 (2.19)	62.88 (2.02)	64.12 (2.06)	61.73 (2.25)
Carbohydrate (g/day)	A	230.67 (7.68)	226.91 (6.70)	221.19 (6.11)	225.10 (6.10)	222.73 (5.89)
	P	224.22 (5.60)	226.64 (6.47)	226.43 (6.17)	227.42 (5.47)	225.02 (5.78)
Dietary fiber (g/day)	A	10.79 (0.47)	9.98 (0.34)^*^	10.41 (0.40)	10.50 (0.42)	10.40 (0.48)
	P	10.56 (0.37)	9.84 (0.43)	10.26 (0.42)	10.37 (0.39)	10.67 (0.44)
Pedometer count (steps/day)	A	7766.4 (410.2)	8098.6 (436.2)	7799.5 (403.3)	8011.9 (398.5)	7980.5 (397.2)
	P	8350.7 (387.4)	8192.9 (403.5)	8254.4 (350.1)	8094.8 (350.2)	8089.6 (348.7)

Data are expressed as means (SEM). A, Active group (*n* = 91); P, Placebo group (*n* = 87). ^*^ *P* < 0.05 *vs.* 0 w

### Primary endpoint

**Fig. 2:**
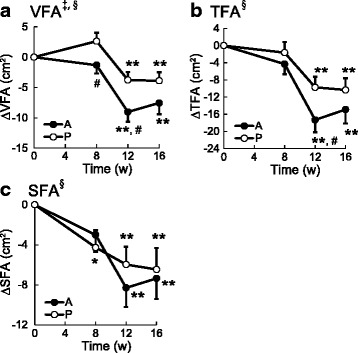
Effects of MHE on abdominal fat areas. Change in VFA (a), TFA (b) and SFA (c). Data are calculated as the degrees of change from the start values at 0 w (∆), and expressed as means (SEM). ^*^ *P* < 0.05, ^**^ *P* < 0.01 *vs*. 0 w (by paired Student’s *t*-test), ^#^ *P* < 0.05 *vs*. placebo group (by unpaired Student’s *t*-test). A, Active group (*n* = 91); P, Placebo group (*n* = 87). There was a significant effect of group (^‡^ *P* < 0.01), and of time (^§^ *P* < 0.01) during the test-beverage ingestion period (by two-way ANOVA)

Figure 2 shows the change from the value at 0 w in visceral, subcutaneous and total abdominal fat areas during the course of test-beverage ingestion (0 to 12 w) and follow-up (12 to 16 w). VFA and TFA had decreased significantly after 12 and 16 w in both groups but were significantly lower in the active group than in the placebo group after 8 w (VFA) and 12 w (VFA, TFA) (*P* < 0.05 at each point for each parameter). No significant difference in SFA was observed between the two groups, although SFA was significantly decreased after 8, 12 and 16 w in the placebo group, and after 12 and 16 w in the active group. Two-way ANOVA indicated a significant effect of group on VFA, significant effects of time on VFA, TFA and SFA, and no significant time-by-group interaction for any parameters during the test-beverage ingestion period. There were no significant differences between the two groups for each of the three abdominal fat areas when test-beverage ingestion began (0 w).

### Secondary endpoints

Table 4 shows the change in secondary endpoints since the start of test-beverage ingestion (0 w). Although secondary endpoints for either group were not significantly different at the beginning (data not shown), the body fat ratio in the active group after 4, 8 and 12 w was slightly but significantly lower than in the placebo group (*P* < 0.05 at each point). Body weight and BMI were significantly lower in the active group after 4 w (*P* < 0.01 for each parameter), and tended to be lower after 12 w (*P* = 0.0697 for body weight and *P* = 0.0644 for BMI), than in the placebo group. Body weight and BMI were significantly decreased after 4, 12 and 16 w since the start of test-beverage ingestion (0 w) in the active group, while no significant variations were observed in the placebo group. In addition, waist circumference and hip circumference were significantly lower in the active group after 4 w than the placebo group (*P* < 0.05 for each parameter). Two-way ANOVA indicated significant effects of group on body weight, BMI and body fat ratio, significant effects of time on waist circumference, hip circumference and the ratio of waist to hip circumferences, and no significant time-by-group interaction for any parameters during the test-beverage ingestion period.

**Table 4:** Effect of MHE on body indices

Parameter	Group	Change from the value at 0 w			
		Ingestion period			Follow-up period
		4 w	8 w	12 w	16 w
∆Body weight^†^ (kg)	A	−0.30 (0.10)^##,**^	−0.21 (0.12)	−0.47 (0.17)^**^	−0.51 (0.18)^**^
	P	0.16 (0.10)	0.07 (0.17)	−0.02 (0.18)	0.00 (0.21)
∆BMI ^†^ (kg/m^2^)	A	−0.11 (0.04)^##,**^	−0.08 (0.04)	−0.17 (0.06)^**^	−0.19 (0.07)^**^
	P	0.06 (0.04)	0.02 (0.06)	−0.01 (0.07)	0.00 (0.08)
∆Body fat ratio ^†^ (%)	A	−0.29 (0.13)^#,*^	−0.20 (0.15)^#^	−0.10 (0.16)^#^	−0.16 (0.17)
	P	0.23 (0.17)	0.28 (0.15)	0.41 (0.20)^*^	0.34 (0.20)
∆Waist circumference ^§^ (cm)	A	−0.52 (0.14)^#,**^	−0.62 (0.17)^**^	−0.97 (0.22)^**^	−1.17 (0.23)^**^
	P	−0.10 (0.10)	−0.26 (0.15)	−0.58 (0.18)^**^	−0.54 (0.27)^*^
∆Hip circumference ^§^ (cm)	A	−0.38 (0.10)^#,**^	−0.50 (0.11)^**^	−0.69 (0.14)^**^	−0.81 (0.19)^**^
	P	−0.12 (0.08)	−0.36 (0.12)^**^	−0.46 (0.14)^**^	−0.48 (0.15)^**^
∆Waist/Hip ^§^	A	−0.002 (0.001)	−0.002 (0.001)	−0.003 (0.002)	−0.004 (0.002)
	P	0.000 (0.001)	0.001 (0.001)	−0.002 (0.001)	−0.001 (0.002)

Data are calculated as the degrees of change from the values at 0 w (∆), and expressed as means (SEM). A, Active group (*n* = 91); P, Placebo group (*n* = 87). ^#^ *P* < 0.05, ^##^ *P* < 0.01 *vs.* Placebo group. ^*^ *P* < 0.05, ^**^ *P* < 0.01 *vs*. 0 w. There was a significant effect of group (^†^ *P* < 0.05), and of time (^§^ *P* < 0.01) during the test-beverage ingestion period (by two-way ANOVA)

### Safety endpoints

Safety endpoint evaluation was conducted on 200 subjects who ingested test beverages at least once (The statistical analyses of parameters for safety endpoint evaluation were conducted on 188 subjects who finished the study.). Table 5 shows the result of circulatory parameter measurement. SBP was significantly increased after 16 w compared to the begin value (0 w) in the placebo group. DBP showed no significant variation from the start value (0 w) in both groups throughout the study. There was a significant difference in pulse rate between the two groups after 8, 12 and 16 w, while the only significant variation from the start value (0 w) within a group was observed after 4 w for the active group: an increase which was not sustained. Comparison of the analysis set for safety endpoint evaluation, with that for secondary endpoint evaluation showed the same body weight variation (data not shown). Body weight and values of all circulatory parameters were within the range of physiological variation.

**Table 5:** Change in circulatory parameters

Parameter	Group	Ingestion period				Follow-up period
		0 w	4 w	8 w	12 w	16 w
SBP (mmHg)	A	119.9 (1.3)	120.7 (1.4)	120.0 (1.2)	119.2 (1.2)	120.5 (1.2)
	P	120.3 (1.2)	120.9 (1.3)	118.9 (1.2)	120.3 (1.3)	122.1 (1.2)^*^
DBP (mmHg)	A	71.2 (1.1)	72.2 (0.9)	71.9 (0.9)	71.7 (1.0)	72.3 (1.0)
	P	72.8 (0.9)	73.3 (1.0)	72.1 (0.8)	72.8 (0.9)	73.5 (1.0)
Pulse rate (bpm)	A	70.0 (0.9)	71.5 (0.9)^*^	69.1 (0.9)^#^	69.9 (0.9)^#^	69.9 (1.0)^#^
	P	72.4 (0.9)	73.3 (0.9)	71.9 (0.8)	73.1 (0.9)	72.8 (0.9)

Data are expressed as means (SEM). A, Active group (*n* = 94); P, Placebo group (*n* = 94)

^#^ *P* < 0.05 *vs.* Placebo group. ^*^ *P* < 0.05 *vs.* 0 w

Tables 6, 7, 8 and 9 show the result of blood chemistry examination. γ-GTP (male), PL, UA (male and female), Cl, Fe (male), UIBC (female) and ferritin (male and female) showed no significant variations from the start values (0 w) in both groups and no significant differences between the two groups. Na and Fe (female) showed significant differences between the two groups, while there were no significant variations from the begin values (0 w) in both groups. TP, Alb, T-Bil, AST, ALT, LDH, ALP, γ-GTP (female), TC, LDL-C, HDL-C (male and female), TG, FFA, glucose, BUN, Cre (male and female), K, Ca, Mg, TIBC (male and female) and UIBC (male) showed some significant variation from the begin values (0 w) in either group, while there were no significant differences between the two groups at any point except for the start value (0 w). Values of all blood chemistry parameters were within reference ranges.

**Table 6:** Change in blood chemistry parameters (liver function)

Parameter	Sex	Reference range	Group	Ingestion period				Follow-up period
				0 w	4 w	8 w	12 w	16 w
TP (g/dL)		6.5-8.2	A	7.30 (0.03)	7.27 (0.04)	7.23 (0.03)^*^	7.26 (0.03)	7.30 (0.03)
			P	7.29 (0.03)	7.22 (0.03)^*^	7.28 (0.04)	7.28 (0.04)	7.31 (0.04)
Alb (g/dL)		3.7-5.5	A	4.46 (0.02)	4.43 (0.02)	4.42 (0.02)^*^	4.43 (0.02)^*^	4.48 (0.02)
			P	4.50 (0.02)	4.46 (0.02)^*^	4.48 (0.02)	4.48 (0.03)	4.54 (0.02)^*^
T-Bil (mg/dL)		0.3-1.2	A	0.70 (0.03)	0.72 (0.03)	0.68 (0.03)	0.68 (0.03)	0.68 (0.03)
			P	0.74 (0.03)	0.72 (0.03)	0.69 (0.03)	0.71 (0.03)	0.67 (0.03)^**^
AST (U/L)		10-40	A	21.1 (0.6)	20.5 (0.6)^*^	21.2 (0.7)	21.5 (0.9)	22.5 (0.8)^*^
			P	20.9 (0.6)	20.7 (0.7)	21.3 (0.9)	21.4 (0.8)	21.4 (0.5)
ALT (U/L)		5-45	A	23.7 (1.3)	22.0 (1.3)^**^	23.0 (1.3)	23.3 (1.6)	25.0 (1.7)
			P	22.2 (1.4)	21.0 (1.1)	24.1 (1.8)	23.5 (1.5)	22.5 (1.0)
LDH (U/L)		120-245	A	172.4 (2.5)	178.9 (2.8)^**^	174.9 (2.9)	179.1 (3.5)^**^	178.8 (3.7)^*^
			P	175.5 (3.6)	179.3 (3.1)	175.6 (3.3)	173.3 (2.7)	174.1 (2.8)
ALP (U/L)		104-338	A	199.4 (5.3)	199.7 (5.4)	198.4 (5.3)	197.8 (5.1)	200.7 (5.4)
			P	190.2 (5.5)	187.7 (5.5)	193.6 (5.6)	195.6 (5.9)^*^	197.5 (6.0)^**^
γ-GTP (U/L)	M	≤79	A	37.7 (3.8)	36.1 (3.6)	37.6 (4.0)	37.0 (4.0)	38.9 (3.9)
			P	39.7 (5.2)	39.6 (4.3)	43.2 (5.6)	41.0 (5.1)	40.7 (3.8)
	F	≤48	A	24.7 (2.2)^#^	23.2 (1.7)	24.8 (2.3)	23.8 (2.0)	26.8 (2.5)
			P	19.7 (1.3)	19.2 (1.2)	21.5 (1.6)^*^	20.7 (1.4)	21.6 (2.0)

Data are expressed as means (SEM). A, Active group (*n* = 94); P, Placebo group (*n* = 94). M, Male; F, Female

^#^ *P* < 0.05 *vs.* Placebo group. ^*^ *P* < 0.05, ^**^ *P* < 0.01 *vs.* 0 w

**Table 7:** Change in blood chemistry parameters (lipids and glucose)

Parameter	Sex	Reference range	Group	Ingestion period				Follow-up period
				0 w	4 w	8 w	12 w	16 w
TC (mg/dL)		150-219	A	205.1 (3.6)	202.8 (3.5)	203.5 (3.5)	203.1 (3.6)	207.4 (3.8)
			P	197.6 (3.7)	194.4 (3.5)	197.1 (3.7)	198.2 (3.7)	200.7 (3.8)^*^
LDL-C (mg/dL)		70-139	A	128.7 (3.3)	126.8 (3.2)	126.6 (3.2)	124.9 (3.3)^*^	128.7 (3.4)
			P	121.5 (3.2)	118.1 (3.2)^*^	121.4 (3.4)	119.6 (3.3)	123.1 (3.4)
HDL-C (mg/dL)	M	40-80	A	52.5 (1.7)	50.1 (1.5)^**^	50.8 (1.6)^*^	52.3 (1.6)	53.9 (1.7)
			P	51.2 (1.6)	50.4 (1.9)	50.1 (1.9)	52.4 (1.9)	54.0 (1.8)^**^
	F	40-90	A	56.4 (1.4)	55.1 (1.4)^*^	55.0 (1.5)	57.2 (1.4)	57.6 (1.5)
			P	55.9 (1.4)	54.3 (1.4)^**^	55.5 (1.5)	57.1 (1.6)	57.3 (1.5)
TG (mg/dL)		50-149	A	106.2 (8.5)	113.0 (8.3)	123.9 (11.9)^**^	113.1 (9.9)	111.4 (8.0)
			P	112.3 (7.7)	116.8 (7.6)	105.9 (5.6)	113.8 (7.0)	112.5 (6.7)
PL (mg/dL)		150-250	A	218.3 (3.2)	215.1 (2.9)	217.1 (3.1)	215.2 (3.2)	219.9 (3.3)
			P	212.5 (3.0)	210.7 (3.0)	209.7 (2.9)	212.0 (3.0)	214.7 (2.8)
FFA (mEq/L)		0.10-0.81	A	0.539 (0.019)	0.625 (0.025)^**^	0.524 (0.021)	0.545 (0.022)	0.563 (0.019)
			P	0.542 (0.021)	0.592 (0.023)	0.545 (0.020)	0.531 (0.019)	0.523 (0.019)
Glucose (mg/dL)		70-109	A	93.4 (0.9)	94.1 (0.9)	93.8 (0.9)	92.4 (0.9)	94.4 (1.0)
			P	93.7 (1.0)	93.2 (1.0)	92.8 (1.0)	92.4 (1.0)^*^	94.6 (1.1)

Data are expressed as means (SEM). A, Active group (*n* = 94); P, Placebo group (*n* = 94). M, Male; F, Female

^*^ *P* < 0.05, ^**^ *P* < 0.01 *vs.* 0 w

**Table 8:** Change in blood chemistry parameters (kidney function and electrolyte)

Parameter	Sex	Reference range	Group	Ingestion period				Follow-up period
				0 w	4 w	8 w	12 w	16 w
UA (mg/dL)	M	3.6-7.0	A	6.37 (0.15)	6.38 (0.17)	6.39 (0.14)	6.43 (0.16)	6.35 (0.16)
			P	6.36 (0.17)	6.40 (0.18)	6.58 (0.17)	6.41 (0.18)	6.29 (0.14)
	F	2.7-7.0	A	5.09 (0.13)	4.96 (0.14)	5.07 (0.12)	4.98 (0.15)	5.05 (0.13)
			P	5.18 (0.14)	5.09 (0.14)	5.18 (0.14)	5.13 (0.14)	5.13 (0.14)
BUN (mg/dL)		8.0-20.0	A	13.65 (0.37)	13.58 (0.37)	13.12 (0.38)	12.67 (0.33)^**^	13.23 (0.30)
			P	13.72 (0.35)	13.28 (0.35)	13.31 (0.38)	12.80 (0.30)^**^	13.32 (0.30)
Cre (mg/dL)	M	0.65-1.09	A	0.816 (0.015)	0.811 (0.014)	0.809 (0.014)	0.791 (0.014)^**^	0.804 (0.013)
			P	0.833 (0.019)	0.828 (0.019)	0.839 (0.020)	0.820 (0.019)	0.826 (0.021)
	F	0.46-0.82	A	0.619 (0.014)	0.610 (0.015)	0.618 (0.015)	0.597 (0.013)^*^	0.619 (0.015)
			P	0.629 (0.014)	0.617 (0.013)	0.625 (0.013)	0.606 (0.012)^*^	0.617 (0.012)
Na (mEq/L)		135-145	A	140.5 (0.2)^##^	140.6 (0.2)	140.5 (0.2)^#^	140.8 (0.2)	140.6 (0.2)
			P	141.3 (0.2)	141.0 (0.2)	141.1 (0.2)	140.9 (0.2)	141.0 (0.2)
Cl (mEq/L)		98-108	A	103.9 (0.2)	103.8 (0.2)	104.0 (0.2)	103.8 (0.2)	103.6 (0.2)
			P	104.1 (0.2)	104.1 (0.2)	104.2 (0.2)	103.9 (0.2)	103.8 (0.2)
K (mEq/L)		3.5-5.0	A	4.18 (0.03)^#^	4.08 (0.02)^**^	4.16 (0.03)	4.14 (0.03)	4.18 (0.03)
			P	4.11 (0.03)	4.05 (0.03)	4.10 (0.03)	4.11 (0.02)	4.16 (0.02)^*^
Ca (mg/dL)		8.6-10.2	A	9.38 (0.03)	9.37 (0.04)	9.34 (0.04)	9.36 (0.03)	9.39 (0.03)
			P	9.41 (0.04)	9.34 (0.04)^*^	9.40 (0.04)	9.41 (0.04)	9.41 (0.03)
Mg (mg/dL)		1.7-2.6	A	2.21 (0.02)	2.21 (0.01)	2.18 (0.01)^*^	2.19 (0.01)	2.19 (0.01)
			P	2.20 (0.02)	2.22 (0.02)	2.21 (0.02)	2.20 (0.02)	2.20 (0.02)

Data are expressed as means (SEM). A, Active group (*n* = 94); P, Placebo group (*n* = 94). M, Male; F, Female

^#^ *P* < 0.05, ^##^ *P* < 0.01 *vs.* Placebo group. ^*^ *P* < 0.05, ^**^ *P* < 0.01 *vs.* 0 w

**Table 9:** Change in blood chemistry parameters (iron metabolism)

Parameter	Sex	Reference range	Group	Ingestion period				Follow-up period
				0 w	4 w	8 w	12 w	16 w
Fe (μg/dL)	M	60-210	A	107.7 (5.0)	116.6 (6.3)	101.6 (4.4)	110.1 (5.4)	112.9 (6.7)
			P	112.7 (5.6)	110.9 (5.6)	102.3 (5.0)	104.5 (5.2)	101.8 (5.0)
	F	50-170	A	94.3 (4.8)	89.8 (4.6)^#^	94.1 (4.5)	86.1 (4.9)^##^	98.0 (4.8)
			P	102.8 (4.9)	103.3 (5.0)	97.7 (5.3)	108.9 (5.3)	98.0 (5.4)
TIBC (μg/dL)	M	250-410	A	323.7 (4.3)	317.7 (5.1)^*^	315.3 (4.2)^**^	319.4 (4.3)^*^	322.8 (4.4)
			P	332.4 (5.4)	327.5 (4.8)^*^	326.2 (5.6)^*^	325.6 (5.3)^*^	329.4 (4.9)
	F	250-460	A	341.7 (6.7)	331.9 (6.0)^**^	335.6 (6.5)^*^	333.3 (6.5)^**^	336.1 (6.3)
			P	334.2 (6.1)	328.5 (5.5)^*^	334.1 (6.5)	332.9 (6.4)	336.7 (6.5)
UIBC (μg/dL)	M	120-330	A	216.0 (5.9)	201.1 (6.8)^*^	213.7 (5.5)	209.3 (5.6)	210.0 (6.5)
			P	219.8 (6.9)	216.7 (7.2)	223.9 (7.6)	221.2 (7.0)	227.6 (6.2)
	F	110-425	A	247.4 (9.0)	242.2 (8.7)	241.5 (9.3)	247.2 (9.3)	238.1 (8.8)
			P	231.3 (8.3)	225.1 (7.6)	236.4 (8.6)	224.0 (9.3)	238.7 (8.7)
Ferritin (ng/mL)	M	21-282	A	147.76 (8.63)	151.39 (9.24)	147.35 (8.80)	146.59 (9.03)	150.44 (9.45)
			P	137.97 (12.51)	142.62 (14.06)	143.55 (13.20)	145.89 (13.65)	143.39 (14.07)
	F	5-157	A	51.71 (6.56)	49.91 (6.23)	50.46 (6.67)	54.47 (7.36)	54.44 (6.82)
			P	48.63 (4.32)	45.86 (4.07)	49.63 (4.30)	51.02 (4.54)	49.55 (4.41)

Data are expressed as means (SEM). A, Active group (*n* = 94); P, Placebo group (*n* = 94). M, Male; F, Female

^#^ *P* < 0.05, ^##^ *P* < 0.01 *vs.* Placebo group. ^*^ *P* < 0.05, ^**^ *P* < 0.01 *vs.* 0 w

Table 10 shows the result of hematological examination. WBC, RBC (male and female), Hb (male and female), Ht (male and female) and Plt showed some significant variations from the begin values (0 w) in either group, but there were no significant differences between the two groups at any point except for 0 w. Values of all hematological parameters were within reference ranges.

**Table 10:** Change in hematological parameters

Parameter	Sex	Reference range	Group	Ingestion period				Follow-up period
				0 w	4 w	8 w	12 w	16 w
WBC (/μL)		3500-9700	A	6090.1 (157.0)	6423.4 (183.2)^**^	6188.5 (166.2)	6298.6 (188.2)	6288.3 (156.4)^*^
			P	5986.3 (143.3)	6094.6 (157.6)	6011.3 (143.5)	6108.6 (156.2)	6230.1 (153.6)^*^
RBC (×10^4^/μL)	M	438-577	A	491.9 (4.7)^#^	489.2 (4.7)	487.6 (4.5)^*^	489.7 (5.1)	495.0 (4.8)
			P	507.8 (4.6)	498.6 (4.8)^**^	500.2 (4.5)^**^	500.1 (4.8)^**^	504.1 (4.9)
	F	376-516	A	449.4 (4.2)	446.7 (4.5)	445.7 (4.2)	449.2 (4.3)	451.6 (4.4)
			P	447.3 (4.4)	444.8 (4.8)	446.8 (4.8)	447.1 (4.7)	452.6 (4.4)^*^
Hb (g/dL)	M	13.6-18.3	A	15.25 (0.13)	15.20 (0.14)	15.18 (0.14)	15.26 (0.17)	15.44 (0.14)^*^
			P	15.51 (0.13)	15.24 (0.14)^**^	15.34 (0.13)^*^	15.32 (0.14)^*^	15.43 (0.14)
	F	11.2-15.2	A	13.36 (0.14)	13.34 (0.14)	13.33 (0.14)	13.41 (0.14)	13.50 (0.14)^*^
			P	13.44 (0.14)	13.38 (0.15)	13.44 (0.15)	13.44 (0.14)	13.60 (0.13)^*^
Ht (%)	M	40.4-51.9	A	45.67 (0.39)	45.59 (0.44)	44.89 (0.43)^**^	45.06 (0.51)	45.66 (0.41)
			P	46.46 (0.38)	45.96 (0.42)^*^	45.12 (0.40)^**^	45.35 (0.33)^**^	45.60 (0.39)^**^
	F	34.3-45.2	A	41.32 (0.36)	40.91 (0.38)	40.66 (0.38)^**^	40.57 (0.31)^**^	40.81 (0.36)^*^
			P	41.38 (0.39)	41.00 (0.42)	40.84 (0.43)^**^	40.63 (0.39)^**^	41.17 (0.37)
Plt (×10^4^/μL)		14.0-37.9	A	25.95 (0.54)	26.06 (0.58)	26.02 (0.54)	25.94 (0.55)	26.74 (0.55)^**^
			P	26.33 (0.59)	26.62 (0.60)	26.67 (0.63)	26.64 (0.59)	27.15 (0.61)^**^

Data are expressed as means (SEM). A, Active group (*n* = 94); P, Placebo group (*n* = 94). M, Male; F, Female

^#^ *P* < 0.05 *vs.* Placebo group. ^*^ *P* < 0.05, ^**^ *P* < 0.01 *vs.* 0 w

No clinically problematic findings were noted in urinalysis throughout the study, whereas urine pH was significantly decreased after 8 w compared to the start value (0 w) in the placebo group within the reference range (Table 11).

**Table 11:** Urinalysis

Parameter	Reference range	Group	Ingestion period				Follow-up period
			0 w	4 w	8 w	12 w	16 w
Urine pH	4.8-7.5	A	5.92 (0.07)	5.81 (0.07)	5.91 (0.07)	5.90 (0.07)	5.79 (0.07)
		P	5.89 (0.06)	5.79 (0.06)	5.73 (0.06)^*^	5.88 (0.06)	5.85 (0.06)

Data are expressed as means (SEM). A, Active group (*n* = 94); P, Placebo group (*n* = 94). ^*^ *P* < 0.05 *vs.* 0 w

During the study period, 83 cases divided over 47 subjects in the active group and 78 cases for 43 subjects in the placebo group were reported as adverse events. Of both groups, the most common adverse event reported related to cold-like symptoms. All adverse events are shown in the Additional file 1. All cases were judged by the site investigators to be mild and to have no relation to the test beverages.

## Discussion

In this study, we investigated the effect of continual MHE ingestion for 12 weeks on reducing body fat in healthy subjects with a BMI of 25 or more and less than 30 kg/m^2^ by an intervention trial without changing their usual lifestyle. Our results show that VFA was significantly decreased in the active group compared to the placebo group after 8 and 12 w. In addition, TFA was also significantly decreased in the active group after 12 w compared to the placebo group. In accordance with the change in abdominal fat area, the body fat ratio was slightly but significantly decreased in the active group compared to the placebo group.

The subjects were instructed to keep their usual eating, exercise, sleeping, smoking, and drinking habits, during the study period. Indeed, energy intake and physical activity did not differ between the two groups throughout the study. As the only difference between the active and placebo beverages was the presence of MHE, the results of our study indicate that ingestion of MHE reduces body fat in healthy subjects with a BMI of 25 to below 30 kg/m^2^, while changes in energy intake and physical activity can be ruled out to have influenced the results.

VFA, TFA and SFA decreased as compared to the baseline value (0 w) not only in the active group but also in the placebo group. The reason for the reduction of VFA, TFA and SFA in the placebo group is unclear at the present time. One possibility is that participation in the study itself may affect the metabolic state of the subjects unconsciously. Indeed, it has been previously reported that the mean values of body fat and body weight in the placebo group decreased as compared to the baseline value (0 w) [23, 24], similar to our study. In addition, seasonal variation may affect VFA, TFA and SFA in the placebo group, because seasonal variations of body weight have been described [25, 26].

Previously, it has been reported that the 12-week minimum effective dose of iso-α-acids needed to reduce VFA significantly as compared to placebo for subjects with prediabetes is 48 mg per day [11]. However, MHE does not contain detectable amounts of iso-α-acids, indicative that the anti-obesity effect of MHE is caused by components other than iso-α-acids. The solid content of the MHE used in this study contained 18.3% of MHBA. We have reported that MHBA ameliorates diet-induced body fat accumulation in rodents [18]. Moreover, studies in rodents suggested that MHBA and MHE with the same amount of MHBA have an equivalent effect on body fat reduction (unpublished data). Hence, we speculate that the effect of MHE on body fat reduction observed in this study is mainly due to MHBA.

Because obesity occurs when energy intake exceeds energy expenditure, inhibition of energy intake or acceleration of energy expenditure is considered to be an important target for preventing obesity [27]. Some food ingredients have been reported to modulate energy intake and energy expenditure in humans, for example, lipid absorption inhibition by indigestible dextrin, and energy expenditure acceleration by capsinoids [28–30]. In addition, bitter tastants have been reported to modulate the energy intake by regulating the appetite *via* ghrelin secretion in mice [31], and by regulating satiation during oral nutrient ingestion *via* altered gastrointestinal motility in humans [32]. In this study, ingestion of MHE did not alter energy intake during the ingestion period, and the time of MHE ingestion was not limited, like at mealtime. These results suggest that the anti-obesity effect of MHE may be induced by energy expenditure acceleration rather than energy intake inhibition, by controlling appetite and lipid absorption. Indeed, we have reported that MHBA enhances thermogenesis in BAT *via* activation of sympathetic nerve activity innervating BAT in rodents [18]. It has also been revealed that adult humans have functional BAT that plays a role in maintaining body weight [20, 21], and BAT activity in humans is controlled by the sympathetic nervous system, as observed in rodents [33]. As one of the food ingredients modulating BAT activity, extract of *Kaempferia parviflora*, a member of the ginger family, has been reported to increase energy expenditure through the activation of BAT in mice and humans [34, 35]. Hence, enhancement of thermogenesis in BAT may contribute to the body fat reduction effect of MHE in this study. On the one hand, MHBA at the concentration in the active beverage in this study (35 mg/350 mL) did not cause a bitter sensation in the oral cavity because its dose was too low to sense bitterness. On the other hand, it has been reported that bitter taste receptors are present not only in taste buds on the tongue but also in endocrine cells of the gastrointestinal tract (GI tract) [36]. On the binding of bitter tastants to bitter taste receptors in the GI tract, hormones or neurotransmitters are released that can communicate with the brain and have a role in the regulation of energy and glucose homeostasis [36]. We have reported that MHBA induces thermogenesis in BAT by oral administration into the gastric cavity in rodents [18]. This suggests that bitter taste receptors in the endocrine cells of the GI tract might be involved in the mechanism underlying the anti-obesity effect of MHBA. In future work, intentional dosing studies will be needed to identify the active components and reveal the anti-obesity mechanism of MHE in detail.

Obesity leads to metabolic disorders such as hypertension, diabetes mellitus and atherosclerosis, which are risk factors for coronary artery disease [1–3]. Many studies have reported that body fat distribution has a close relationship with the occurrence of metabolic disorders, and an excess of abdominal fat, especially intra-abdominal visceral fat, leads to these disorders, irrespective of body weight [37–39]. Accordingly, it is very important to reduce body fat, especially visceral fat for the prevention of metabolic disorders. Our results indicate that MHE reduces TFA by reducing mainly VFA. Therefore, MHE may be useful for preventing obesity as well as reducing the risk of metabolic disorders and coronary artery disease. Adipomyokines such as leptin, adiponectin and irisin, and gut hormones such as ghrelin and glucagon-like peptide-1, are known to play an important role in energy homeostasis [40, 41]. It is presumed that an imbalance in the circulating levels of some of these hormones is related to metabolic disorders [40, 42], and the beneficial effect of food intervention on these profiles has been reported. For example, it has been reported that continual ingestion of pistachio nuts and catechin increase adiponectin level in, respectively, subjects with metabolic syndrome and subjects with type 2 diabetes [43, 44]. Intensive studies on whether MHE affects these hormone levels will provide valuable information concerning the effect of MHE on metabolic disorders.

At the doses used in this study, MHE did not induce abnormal changes in hepatic function, renal function or electrolytes. Body weight and values of all circulatory parameters were within the range of physiological variation, and values of all parameters for blood chemistry, hematology and urinalysis were within the reference ranges. All adverse events reported during the study period were judged to be mild and to be unrelated to the test beverages by the site investigators. Thus, it may be assumed that continual ingestion of MHE for 12 weeks is safe. In addition, the effects of MHE on reducing VFA, TFA and body fat ratio were mitigated at the end of the follow-up period, indicating that continual ingestion of MHE is necessary in order to maintain the anti-obesity effects of MHE.

A potential limitation of the study was the relative short duration, so that any long-term effects of MHE ingestion remain unknown. As the difference in VFA and TFA between the active and placebo groups increased over time and did not plateau by the end of the period of test-beverage ingestion, it is possible that the benefits offered by MHE would increase over a longer period of ingestion.

## Conclusions

This study revealed that continual ingestion of MHE reduces body fat in healthy humans with a BMI of 25 or more and less than 30 kg/m^2^ without the need for any lifestyle change. Intake of MHE (35 mg as the amount of MHBA) per day was sufficient to achieve this effect. Therefore, MHE could be a useful and safe tool to prevent obesity and related metabolic disorders.

## Additional file

Additional file 1: Adverse events. (PDF 83 kb)

## Abbreviations

MHE: matured hop extract
MHBA: matured hop bitter acids
VFA: visceral fat area
TFA: total fat area
SFA: subcutaneous fat area
BMI: body mass index
