# Supplementary material for: Matured hop extract reduces body fat in healthy overweight humans: a randomized, double-blind, placebo-controlled parallel group study
Source: Nutr J. 2016 Mar 9;15:25. doi: 10.1186/s12937-016-0144-2 (PMC4784395; doi:10.1186/s12937-016-0144-2)
Supplement: Additional file 1: — Adverse events. (PDF 83 kb) [file 12937_2016_144_MOESM1_ESM.pdf]

Additional file 1. Adverse events.

Adverse events reported during the study period were as follows: in the active group, 25 cases of cold-like symptoms, 9 cases of headache, 3 cases of diarrhea, 3 cases of sore throat, 3 cases of low back pain, 3 cases of stomachache, 2 cases of tiredness· headache, 2 cases of leaning stomach, 1 case each of chill, hangover, diarrhea· fever, dizziness· headache, calf pain, heartburn, toothache, side ache, scalp eczema, strained back, stomachache· headache, headache· nausea, thenar pain· instep pain, shoulder pain, uncomfortable feeling of back, edema, vomiting· diarrhea, feeling of dyspnea, dysorexia, dysphoria, foot fracture, epigastralgia, erythema multiforme, shoulder pain· neck ache, bruise, stomachache· gastrorrhea, transmissible gastroenteritis, stomatitis, food poisoning, sprain, pituita, itch of face· itch of upper arms, and feeling of fullness; in the placebo group, 20 cases of cold-like symptoms, 7 cases of diarrhea, 6 cases of stomachache, 6 cases of headache, 5 cases of tiredness, 3 cases of edema, 2 cases of heartburn, 2 cases of toothache, 1 case each of jaw pain, pituita, dysorexia, nasal obstruction, headache· stiffness of shoulder, headache· nausea, low back pain· gonalgia· stomachache, gastroenteritis, swelling of the cheek, glow· edema, fever· headache, hangover, backache· shoulder pain· low back pain, eyestrain· low back pain, neck ache, nettle rashhives, headache· dizziness, stomatitis, hordeolum, cystitis, headache· epigastralgia, dysorexia· tiredness, allergic rhinitis, itch of eye, eye pain, low back pain, knee pain· shin pain, swelling.
